# Low-Dose Celastrol Modulates IL-6 Secretion to Overcome Resistance to Sorafenib in Hepatocellular Carcinoma Cells

**DOI:** 10.5812/ijpr-160042

**Published:** 2025-08-05

**Authors:** Rui Zhang, Lingchun Kong, Hezhao Zhang, Anhong Zhang, Zhiyong Shi, Pei Wei

**Affiliations:** 1 The First Hospital of Shanxi Medical University, Taiyuan, China; 2 The Second Hospital of Shanxi Medical University, Taiyuan, China; 3 Department of Immunology, Zhuhai Campus of Zunyi Medical University, Zhuhai, China

**Keywords:** Celastrol, Sorafenib, Drug Resistance, Interleukin-6

## Abstract

**Background:**

Hepatocellular carcinoma (HCC) is a leading cause of cancer-related mortality, with sorafenib being a key treatment option. However, resistance to sorafenib often develops, limiting its effectiveness. Celastrol, a phytochemical derived from *Tripterygium wilfordii*, has shown potential in enhancing anti-tumor drug efficacy, but concerns about toxicity and clinical applicability remain.

**Objectives:**

This study investigated whether celastrol at plasma-achievable concentrations could modulate sorafenib resistance in HCC cells in-vitro.

**Methods:**

Cytotoxicity experiments were conducted using MTT assays to assess the effects of celastrol and sorafenib on HCC cells and normal hepatocytes. Immunofluorescence (IF) and ELISA assays were employed to measure IL-6 expression and secretion in HCC cells. Bioinformatics analyses were performed on publicly available gene expression data to identify pathways associated with sorafenib resistance. Conditioned media (CM) from treated cells were used to evaluate the impact of celastrol on sorafenib sensitivity in untreated HCC cells.

**Results:**

High concentrations of celastrol enhanced sorafenib’s inhibitory effects on HCC cells but also increased cytotoxicity in normal hepatocytes. Low concentrations of celastrol mitigated sorafenib-induced tumor cell inhibition but reversed acquired sorafenib resistance without increasing cytotoxicity in normal hepatocytes. The reversal of resistance by low-dose celastrol was associated with the inhibition of sorafenib-induced IL-6 secretion. The CM from tumor cells treated with low-dose celastrol plus sorafenib increased the sensitivity of untreated tumor cells to sorafenib, an effect reversed by the addition of exogenous IL-6 or by using IL-6-neutralizing antibodies.

**Conclusions:**

Low-dose celastrol can reverse sorafenib resistance in HCC cells by inhibiting sorafenib-induced IL-6 secretion, without increasing hepatotoxicity.

## 1. Background

Hepatocellular carcinoma (HCC), a formidable oncological challenge, is responsible for over 800,000 deaths annually and ranks as the fourth leading cause of cancer-related mortality worldwide (1, 2). Characterized by aggressive growth and poor prognosis, HCC often evades early detection, resulting in advanced-stage presentations that leave patients with limited and frequently ineffective treatment options (1, 2). The advent of sorafenib, a tyrosine kinase inhibitor targeting Raf kinases and receptor tyrosine kinases such as VEGFR and PDGFR, signifies a pivotal milestone in therapeutic interventions (3, 4). By undermining tumoral vascularization and hindering proliferative pathways, sorafenib has extended the survival of patients with late-stage HCC. Nevertheless, its benefits are transient, and resistance to this drug inevitably ensues within a few months of initiation (5, 6). Multiple, often overlapping mechanisms have been implicated in this resistance. At the molecular level, compensatory activation of alternative pro-survival signaling cascades — including PI3K/AKT/mTOR, JAK/STAT3, and Wnt/β-catenin pathways — promotes cell proliferation and survival in the face of RAF/MEK/ERK inhibition (7). Cellular processes such as epithelial-mesenchymal transition (EMT), enhanced autophagy, and evasion of apoptosis further enable HCC cells to withstand sorafenib-induced stress (8, 9). In addition, tumor microenvironmental factors (such as hypoxia), epigenetic reprogramming, and non-coding RNAs have been shown to sustain or even reinforce the resistant phenotype (9, 10).

Drug resistance has urged the search for novel strategies to reinforce antitumoral drugs against HCC. Phytochemicals — bioactive compounds extracted from plants — have emerged as promising substances (11, 12). Their multimodal mechanisms, low toxicity profile, and capacity to modulate resistance pathways render them prime candidates for adjunct therapies that enhance the efficacy of conventional drugs such as sorafenib (13, 14). Among these phytochemicals, celastrol is a diterpenoid epoxide isolated from the Chinese medicinal herb *Tripterygium wilfordii* and has garnered attention for its synergistic effects with sorafenib (13, 14). This compound augments sorafenib’s proapoptotic and antiproliferative effects on HCC cells in-vitro by modulating cell stress and survival pathways (15, 16). However, concerns about its clinical applicability have arisen because the concentrations of celastrol used in current research methods are several orders of magnitude higher than what can be achieved in human plasma (17-19). One of these concerns is the delicate balance between the efficacy and toxicity of *Tripterygium*-derived medications (17-19).

These compounds are notorious for their Narrow Therapeutic Index, necessitating precise dosing to avoid crossing the fine line into toxicity. Elevated levels of celastrol could lead to severe hepatic injury — an extensively documented consequence (18, 19). Importantly, although high-dose celastrol has been shown to synergize with sorafenib to enhance direct tumor cell killing (15, 16), such concentrations far exceed clinically achievable plasma levels. Furthermore, while IL-6 has been identified as a key mediator of both intrinsic and acquired sorafenib resistance in HCC (7), no study to date has evaluated whether a clinically attainable dose of celastrol can modulate sorafenib-induced IL-6 secretion to prevent or reverse resistance. Here, we, for the first time, demonstrate that low-dose celastrol (≤ 0.2 μM), within the range of reported human plasma concentrations, functions as a “resistance shield” by specifically suppressing sorafenib-elicited IL-6 release, thereby both preventing the onset of and reversing established sorafenib resistance in HCC cells — without incurring additional hepatocyte toxicity. This novel approach offers a safer, more sustainable combination strategy to prolong sorafenib efficacy in advanced HCC therapy.

## 2. Objectives

This study investigated whether celastrol at plasma-achievable concentrations could modulate sorafenib resistance in HCC cells in vitro.

## 3. Methods

### 3.1. Reagents

The following reagents and materials were used in this study: Fetal bovine serum (FBS, Inner Mongolia Opcel Biotech, Helingeer, China); MTT kit (Keygenbio, Nanjing, China); celastrol (Tao Su Biotech Co., Ltd., Shanghai, China); sorafenib (Abmole, Shanghai, China); human IL-6 ELISA kit and rabbit anti-human IL-6 antibody (Abcam, Shanghai, China); AKT activator SC79, phosphorylated AKT antibody, total AKT antibody, and human IL-6 neutralizing antibody (R&D System, MN, USA).

### 3.2. Cytotoxicity Experiments

Cytotoxicity to tumor cells and normal hepatocytes was determined by MTT assays. The cells were cultured in a 96-well plate at the optimized density of 0.6 × 10^5^ per well for 24 h and then treated with the diluted drugs at various concentrations for the indicated times. Each well was added with 10 µL of MTT (5 mg/mL). After 4 h of incubation, the mixed medium was replaced with 150 µL of dimethyl sulfoxide, and absorbance was detected by a microplate reader at a wavelength of 490 nm.

### 3.3. Immunofluorescence

The immunofluorescence (IF) assay was used to detect IL-6 expression in human HCC cells. The cells were fixed with 4% paraformaldehyde for 15 min at room temperature, permeabilized with 0.5% Triton X-100 for 5 min, and blocked with 5% bovine serum albumin for 1 h at room temperature. The cells were then incubated with a specific anti-IL6 antibody overnight at 4°C. After being washed, the cells were added with a secondary antibody conjugated to Alexa Fluor 549 and incubated for 1 h at room temperature. The cells were then washed again and mounted with DAPI for nuclear staining. The samples were visualized under a fluorescence microscope, and images were captured using a digital camera.

### 3.4. Bioinformatics Analyses

The original data of GSE225537 were downloaded from the public Gene Expression Omnibus (GEO, https://www.ncbi.nlm.nih.gov/geo/query/acc.cgi?acc = GSE225537). Differential gene expression (DGE) analysis was performed on the count data using the R package DESeq2, and gene set enrichment analysis (GSEA) was implemented by GSEA v4.3.2.

### 3.5. ELISA Assays

The tumor cells were treated as described above for 48 h. The IL-6 levels in the supernatants were measured using an ELISA kit according to the user manual and previous descriptions (20). Absorbance was measured with a microplate reader (BioTek Instruments, USA) at 450 nm, and the IL-6 content was calculated against the standards and normalized to cell number.

### 3.6. In-cell Western

After treatment, cancer cells were fixed, permeabilized, and blocked. The cells were then incubated with phosphorylated AKT antibody and total AKT antibody (phosphorylated AKT antibody, dilution 1:400; total AKT antibody, dilution 1:600). After washing and incubating with IRDye^®^ 800CW Donkey anti-Rabbit IgG (for phosphorylated AKT antibody) and IRDye^®^ 680RD Donkey anti-Mouse IgG (for total AKT antibody), plates were then scanned using a LI-COR Odyssey CLx Infrared Imaging System.

### 3.7. Statistical Analysis

The data were shown as mean ± standard deviation of triplicates. Statistical comparisons were performed by ANOVA. A P-value of < 0.05 was considered statistically significant.

## 4. Results

### 4.1. Bidirectional Modulation of Sorafenib-Induced HepG2 Cell Inhibition by Celastrol at Varied Concentrations

**Figure 1.**
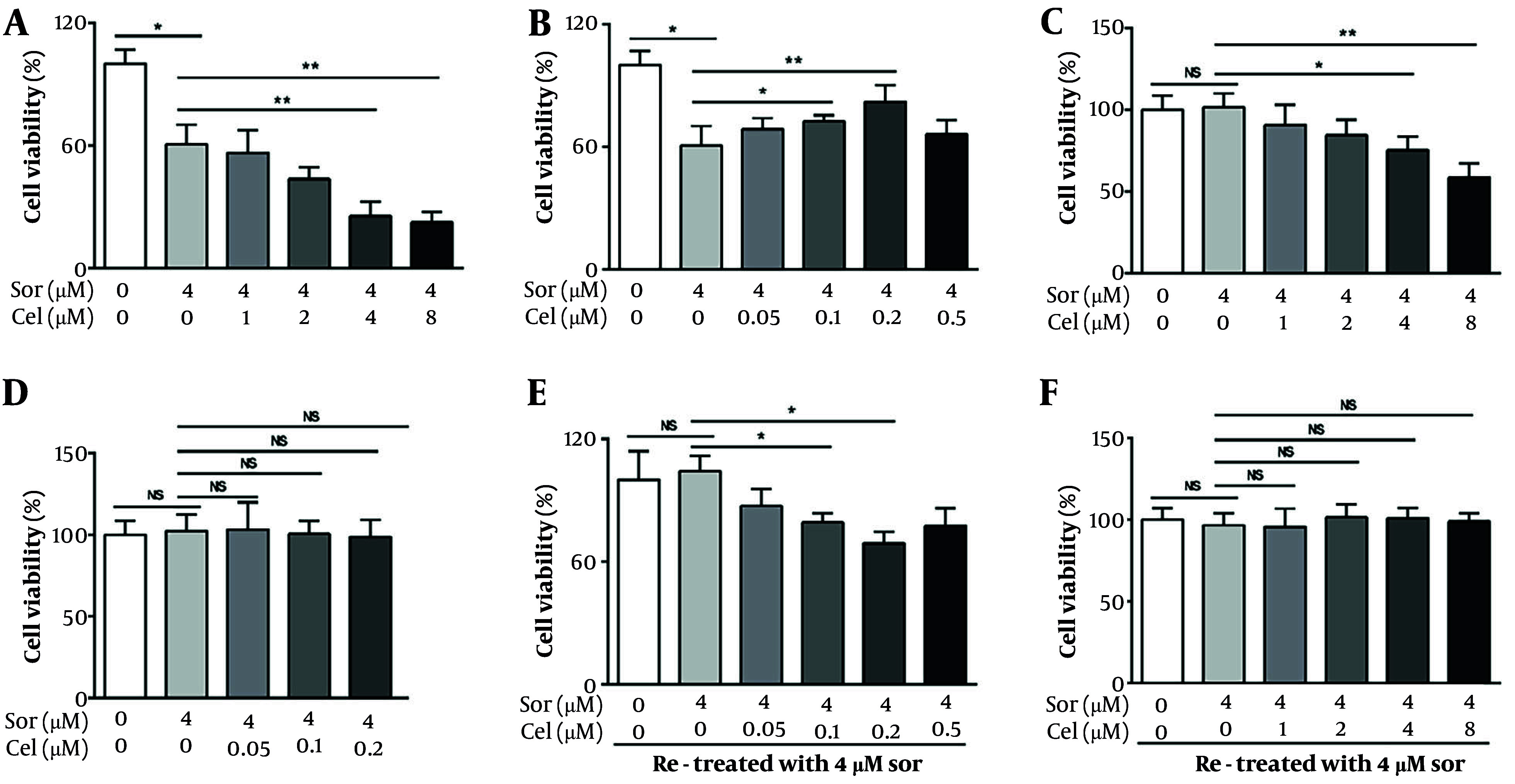
Bidirectional modulation of sorafenib-induced HepG2 cell inhibition by celastrol at varied concentrations. A, tumor cells were exposed to sorafenib alone or in combination with various high concentrations of celastrol (1, 2, 4, or 8 μmol/L) for 72 h before being subjected to MTT assay. At concentrations greater than 2 μmol/L, celastrol enhanced the sorafenib-induced cytotoxicity to hepatocellular carcinoma (HCC) cells; B, tumor cells were exposed to sorafenib alone or in combination with low concentrations of celastrol (0.05, 0.1, 0.2, or 0.5 μmol/L) for 72 h before being subjected to MTT assay. Celastrol dose-dependently inhibited the sorafenib-induced cytotoxicity to HCC cells; C, LO_2_ cells were exposed to sorafenib alone or in combination with high concentrations of celastrol (1, 2, 4, or 8 μmol/L) for 72 h before being subjected to MTT assay. At concentrations greater than 4 μmol/L, celastrol enhanced the cytotoxicity induced by sorafenib toward LO_2_ cells; D, LO_2_ cells were exposed to sorafenib alone or in combination with low concentrations of celastrol (0.05, 0.1, or 0.2 μmol/L) for 72 h before being subjected to MTT assay. Sorafenib was not cytotoxic when administered alone as in combination with celastrol; E, tumor cells were pretreated with 0.4 μm sorafenib for 48 h, washed twice, and retreated with 0.4 μm sorafenib for another 48 h; F, tumor cells were pretreated with 0.4 μm sorafenib and celastrol (0.2 or 8 μm) for 48 h, washed twice, and retreated with 0.4 μm sorafenib for another 48 h. MTT assays were tested to evaluate the cytotoxic effects in HepG2 cells (Abbreviations: Sor, sorafenib; cel, celastrol; NS, not significant. *P < 0.05, **P < 0.01).

In clinical practice, the recommended dose of sorafenib is 400 or 200 mg/twice/day, corresponding to a blood concentration of approximately 0.8 - 4 µM (21). Hence, we selected 4 µM as the representative working concentration of sorafenib for subsequent in vitro experiments. Based on the concentrations of celastrol used in previous studies (22, 23), we define a high concentration bracket for celastrol in this study as 2 - 8 µM. Furthermore, oral celastrol administration reportedly yields plasma levels of around 0.05 - 0.2 µM (24), which we adopted as the low-concentration range for our investigations. As delineated by cytotoxic assays, our data indicate that at elevated concentrations (≥ 2 µM), celastrol amplified sorafenib's growth-inhibitory impact on HepG2 cells (Figure 1A). Low concentrations of celastrol (0.1 and 0.2 µM) unexpectedly mitigated the suppressive effects of sorafenib on the proliferation of HepG2 cells (Figure 1B). We also assessed the potential modulatory effects of celastrol on the cytotoxicity induced by sorafenib to normal hepatic cells LO_2_. Consistent with prior studies, sorafenib at its operational concentration of 4 µM did not exhibit cytotoxicity toward LO_2_ hepatocytes. However, upon administering high concentrations of celastrol (≥ 4 µM) alongside sorafenib, we observed a significant enhancement in cytotoxicity against LO_2_ cells (Figure 1C). After assessing the combined effect of low concentrations of celastrol (ranging from 0.05 µM to 0.5 µM) with sorafenib on LO_2_ cells, we detected no substantial increase in cell death (Figure 1D). 

We further investigated whether different celastrol concentrations could antagonize the acquisition of sorafenib resistance in HCC cells. HepG2 cells were pretreated with 4 µM sorafenib for 24 h, washed twice with serum-free medium, and treated with the same concentration of sorafenib. We observed that the HepG2 cells exhibited insensitivity to the rechallenge with sorafenib, indicating an acquired resistance mechanism. In parallel experiments, we introduced low concentrations of celastrol in conjunction with the sorafenib pretreatment. This combination restored the sensitivity of HepG2 cells to the subsequent sorafenib challenge, remarkably reducing the cell viability compared with that in the sorafenib-only treated group (Figure 1E). Such reversal of resistance was not witnessed when high concentrations of celastrol were used. These results suggest that low-concentration celastrol may modulate the key pathways involved in sorafenib resistance, enhancing the therapeutic vulnerability of HCC cells to sorafenib retreatment.

### 4.2. Low-Concentration Celastrol Reversed Sorafenib Resistance by Altering the Secretory Profile of HepG2 Cells

**Figure 2.**
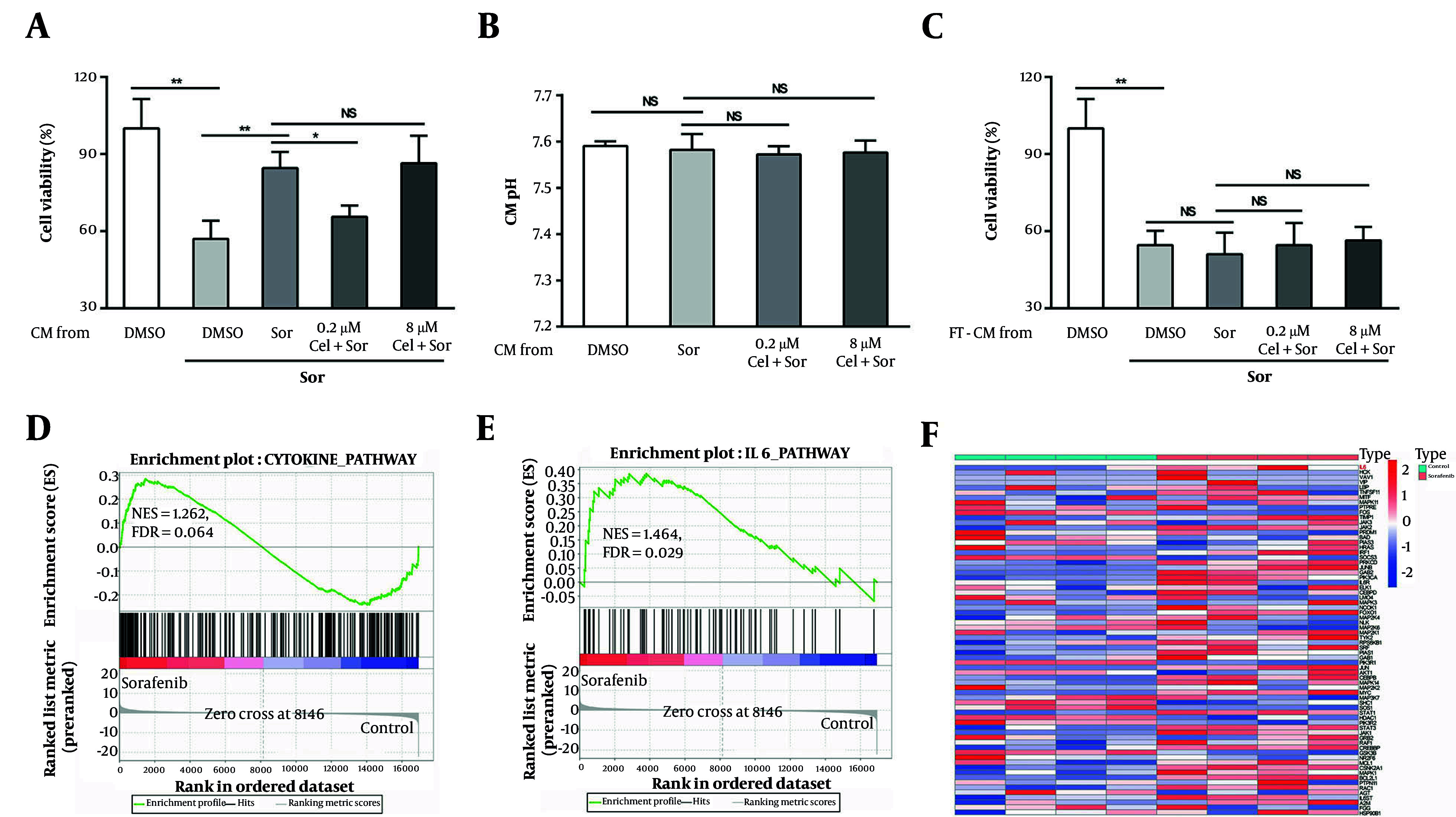
Low-concentration celastrol reversed sorafenib resistance by altering the secretory profile of HepG2 cells. A, effects of various conditioned media (CM) on tumor cell viability after exposure to 0.4 μm sorafenib for 48 h; B, pH of different CM was measured using a pH meter; C, effects of various CM subjected to repeated freeze-thawing on tumor cell viability after exposure to 0.4 μm sorafenib for 48 h. Gene set enrichment analysis (GSEA) results demonstrated that the pathways enriched included the cytokine-biosynthetic process; and E, IL6 signaling pathway; F, the volcano plot, based on Gene Expression Omnibus (GEO) data, showed the up-regulated expression level of IL6 in the sorafenib-treated HepG2 cells (abbreviations: Sor, sorafenib; cel, celastrol; NS, not significant. *P < 0.05, **P < 0.01).

Tumor cells can promote drug resistance by secreting a plethora of protein factors. We hypothesized that the counteraction of HCC cell resistance by low-concentration celastrol may be linked to changes in this secretory behavior. We prepared conditioned media (CM) from HCC cells treated with different concentrations of celastrol plus sorafenib and subsequently cultured untreated HepG2 cells in this CM before challenging them with sorafenib. Our observations revealed that the CM from the cells treated with low-concentration celastrol plus sorafenib significantly increased the sensitivity of HepG2 cells to sorafenib compared with that of the control group (Figure 2A). This sensitization effect was absent in the CM derived from the cells treated with high-concentration celastrol plus sorafenib (Figure 2A). To rule out the possibility that the celastrol-induced changes in CM pH contribute to the observed modulation of drug resistance, we measured the pH values of all CM groups and found no significant differences (Figure 2B). To validate the hypothesis that low-concentration celastrol influences drug resistance by altering the sorafenib-induced secretory profile of tumor cells, we subjected the CM to three freeze-thaw cycles to denature their proteins. Post freeze-thaw treatment, the CM from the low-concentration celastrol plus sorafenib group lost its capacity to enhance the sensitivity of HepG2 cells to sorafenib (Figure 2C). To further validate our hypothesis, we employed bioinformatics analyses to examine the transcriptomes in public databases, comparing control HepG2 cells with sorafenib-treated HepG2 cells. The GSEA revealed a significant enrichment of cytokine-mediated signaling pathways and IL-6 signaling pathways in the sorafenib-treated HCC cells (Figure 2D and E). Additionally, DGE analysis showed a significant upregulation of IL-6 in the sorafenib-treated tumor cells (Figure 2F). These results suggest that low concentrations of triptolide might modulate the resistance of HCC cells to sorafenib by altering the cytokine profile secreted in response to sorafenib treatment, including the upregulation of IL-6.

### 4.3. IL-6-Modulated Resistance of Hepatocellular Carcinoma Cells to Sorafenib

**Figure 3.**
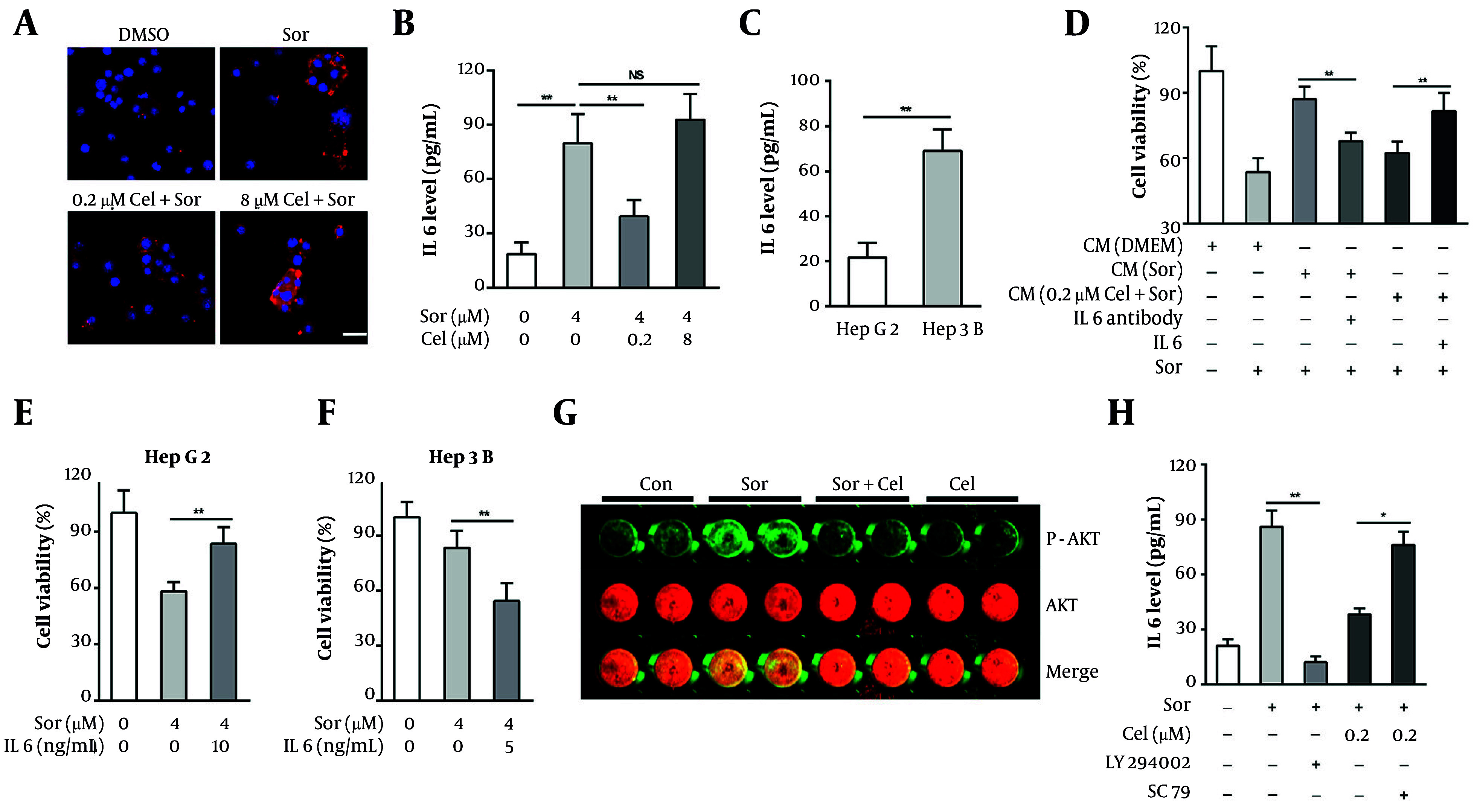
IL6 modulated the resistance of hepatocellular carcinoma (HCC) cells to sorafenib. A, tumor cells were treated with 0.4 μm sorafenib and celastrol (0.2 or 8 μm) for 24 h. The IL6 levels in the tumor cells were then determined via immunofluorescence (IF) assays (red: IL6, blue: DAPI, bar = 20 μM); B, tumor cells were treated with 0.4 μm sorafenib and celastrol (0.2 or 8 μm) for 48 h. The IL6 levels in the supernatants were then determined via ELISA assays; C, the IL6 levels in HepG2 and Hep3B cell supernatants were determined via ELISA assay; D, effects of various conditioned media (CM) containing an IL6-neutralizing antibody (1 μg/mL) or exogenous human IL6 (10 ng/mL) on HepG2 cell viability after exposure to 0.4 μm sorafenib for 72 h; E, HepG2 cells were cotreated with 0.4 μm sorafenib and exogenous human IL6 (10 ng/mL) for 72 h, and the cell viability was tested via MTT assay; F, Hep3B cells were cotreated with 0.4 μm sorafenib and an IL6 neutralizing antibody (1 μg/mL) for 72 h, and the cell viability was tested via MTT assay; G, the levels of phosphorylated and total AKT in HepG2 cells after exposure to 0.4 μm sorafenib and 0.2 μm celastrol for 12 h were determined via In-Cell-Western assay; H, effects of LY294002 (1 μg/mL) or SC79 (2 μM) on IL-6 secretion in HepG2 cells after exposure to 0.4 μm sorafenib and 0.2 μm celastrol for 24 h (abbreviations: Sor, sorafenib; cel, celastrol; NS, not significant. *P < 0.05, **P < 0.01).

IL-6 has been implicated in mediating resistance to a variety of targeted therapies in HCC cells, including sorafenib. We posited that low-concentration celastrol may combat the emergence of resistance by inhibiting sorafenib-induced IL-6 secretion in HCC cells. To test this hypothesis, we evaluated IL-6 expression in HCC cells using an IF assay and quantified its secretion through ELISA. Our observations indicated that sorafenib significantly induced the expression and secretion of IL-6 in HepG2 cells (Figure 3A and B). Meanwhile, celastrol at a low concentration (0.2 µM) significantly inhibited the expression and secretion of IL-6 induced by sorafenib, whereas a high concentration (8 µM) of celastrol did not exhibit this inhibitory effect (Figure 3A and B). Considering that Hep3B cells are less sensitive to sorafenib compared with HepG2 cells, we next compared the IL-6 secretion levels between these two HCC cell lines. The result revealed that IL-6 secretion in Hep3B cells was approximately 3.2-fold higher than in HepG2 cells (Figure 3C), suggesting that IL-6 oversecretion is an important reason why HCC cells resist sorafenib treatment.

To ascertain the role of IL-6 in modulating the resistance of HCC cells to sorafenib, we employed exogenous IL-6 and neutralizing antibodies against IL-6 in our interventions. We observed that the sensitizing effect of the CM from the HepG2 cells treated with low-concentration celastrol plus sorafenib was partially reversed by the addition of exogenous IL-6 (Figure 3D). Meanwhile, the CM from the HCC cells exposed solely to sorafenib with IL-6-neutralizing antibodies enhanced the sensitivity of untreated HepG2 cells to sorafenib (Figure 3D). Moreover, exogenous IL-6 addition counteracted the growth inhibitory effects of sorafenib on HepG2 cells (Figure 3E), and the application of IL-6-neutralizing antibodies increased the sensitivity of Hep3B cells to sorafenib (Figure 3F). These findings collectively elucidate that IL-6 regulates the resistance of HCC cells to sorafenib, and low-concentration celastrol may counteract the emergence of drug resistance by suppressing sorafenib-induced IL-6 secretion. To investigate how celastrol inhibits IL-6 secretion, we focused on the PI3K/AKT pathway — previously shown to be suppressed by celastrol in HCC cells and known to drive sorafenib resistance. In-Cell Western analysis revealed that sorafenib markedly increased AKT phosphorylation without affecting total AKT levels, and that this phosphorylation was attenuated by low-dose celastrol (Figure 3G). ELISA assays further showed that pharmacological inhibition of PI3K/AKT significantly reduced sorafenib-induced IL-6 release, whereas the addition of AKT activator SC79 restored IL-6 secretion despite celastrol treatment (Figure 3H). Together, these results indicate that low-dose celastrol downregulates IL-6 secretion through inhibition of the PI3K/AKT signaling axis.

## 5. Discussion

The complex mechanisms underlying tumor resistance pose significant challenges in cancer therapy. Celastrol, a compound derived from *T. wilfordii* Hook, has shown the ability to enhance the targeted treatment of HCC (13-15). However, the blurred line between its therapeutic efficacy and potential toxicity remains a bottleneck to its clinical deployment. Our findings unveil the potential of low-dose celastrol to resolve sorafenib resistance in HCC treatment.

The concentration-dependent bidirectional regulation of celastrol on sorafenib’s effectiveness in HCC cells underscores the importance of dosage in the clinical application of combination therapies. Although high concentrations of celastrol synergize with sorafenib to bolster the latter’s antiproliferative action, they concurrently elevate the drug’s toxicity toward normal hepatic cells — an unexpected consequence that cautions against simplistic dose escalation strategies for broadening therapeutic effects. Meanwhile, low-dose celastrol did not increase sorafenib’s hepatotoxicity but significantly inhibited its killing effect on HCC cells. This outcome alerts us to the trade-off between avoiding liver toxicity and enhancing sorafenib’s anti-HCC activity under a backdrop where simple dose increases are not feasible. The dual impact of celastrol on sorafenib’s antiproliferative actions in HCC cells underscores the quintessential balance between enhancing efficacy and avoiding toxicity — a tightrope feature of tumor pharmacotherapy.

Our study highlights the strategic utilization of low-dose celastrol as a countermeasure against sorafenib resistance in HCC cells, a capability not afforded by high doses of the compound. This distinction is critical because the clinical plasma levels of celastrol correspond with these low, nontoxic dosages. Rather than enhancing direct antitumor activity, the ability of celastrol to combat the emergence of sorafenib resistance may represent its most valuable contribution to the clinical management of HCC. Our findings reposition celastrol within the oncopharmacology arsenal as a shield, rather than a sword, which contrasts with many previous studies (25-28). The application of low concentrations of celastrol can reverse the acquired sorafenib resistance in HCC cells, indicative of a mechanism that modulates cellular stress responses and survival pathways. Given the widespread clinical challenge presented by therapeutic resistance, this discovery holds significant implications for improving the durability and efficacy of treatments for HCC.

Our exploration into the secretory changes induced by low-dose celastrol treatment reveals the molecular basis of its regulatory role. Analyses of the CM collected from cells treated with low-dose celastrol and sorafenib and verification through protein denaturation highlight the importance of the altered protein secretion patterns modulated by low-dose celastrol. By altering the protein secretion profile induced by sorafenib, low-dose celastrol circumvents traditional cellular barriers that impede drug response. Our data show that low-dose celastrol can inhibit the secretion of IL-6, corroborating the role of this cytokine as a key mediator of resistance in HCC. Given the extensive links established between IL-6 signaling and malignant cell survival under therapeutic stress (29, 30), the inhibition of IL-6 by celastrol appears to be a plausible mechanism through which this phyto-derived agent exerts its sensitizing effect.

Mechanistic studies revealed that low-dose celastrol attenuates sorafenib-induced PI3K/AKT pathway activation, and pharmacological inhibition of the PI3K/AKT pathway phenocopies its suppression of IL-6 secretion, whereas AKT activator SC79 restores IL-6 release. These findings implicate the PI3K/AKT axis as a key mediator through which celastrol reshapes the tumor secretome and overcomes sorafenib resistance.

Our finding that inhibiting sorafenib-induced IL-6 secretion is key to reversing resistance aligns with and extends a substantial body of recent research that has solidified the IL-6/STAT3 axis as a critical mediator of HCC chemoresistance. Recent studies consistently demonstrate that this pathway is not only activated in sorafenib-resistant cells but is also instrumental in maintaining cancer stem cell properties and fostering a resistance-permissive inflammatory tumor microenvironment (31). For instance, Dai et al. identified a feed-forward loop where the stress-induced transcription factor ATF3 upregulates the IL-6 receptor, further amplifying resistance signaling (32).

Concurrently, the field has explored other diverse strategies to circumvent sorafenib tolerance. These include the therapeutic induction of ferroptosis with natural compounds like tiliroside or artesunate, and the complex modulation of protective autophagy (33, 34). Our approach, however, offers a distinct advantage. While many strategies, including those using phytochemicals like berbamine to directly inhibit STAT3, focus on blocking the downstream consequences of pro-survival signaling (35), our use of low-dose celastrol targets the very inception of this resistance loop — the drug-induced secretion of IL-6. This “cytokine-shielding” mechanism, achieved at clinically relevant, non-toxic concentrations, represents a proactive strategy to prevent the establishment of a resistant state, rather than a reactive effort to overcome it once it is established.

Despite these promising findings, our study is not without limitations. First, the experimental models used, primarily in vitro HepG2 and LO_2_ cell lines, may not fully capture the complexity of tumor microenvironment interactions and the heterogeneity of HCC in patients. Translation of these findings to in vivo models and ultimately to clinical practice necessitates cautious interpretation and further validation. Second, although we have identified IL-6 as a key mediator of sorafenib resistance, the broad network of signaling pathways involved remains to be fully elucidated. The interplay among various cytokines, growth factors, and intracellular signaling cascades might have a significant role in dictating cell sensitivity to treatment, suggesting that our understanding of this regulatory network remains incomplete.

Furthermore, the pharmacokinetics and pharmacodynamics of celastrol and sorafenib, particularly when combined with each other, were not extensively explored. Such analyses are crucial for optimizing dosing regimens and minimizing potential adverse effects, especially considering celastrol’s narrow therapeutic window. Nonetheless, the significance of our findings resides in their implications for devising safe and effective therapeutic strategies. By demonstrating that low-dose celastrol can mitigate sorafenib resistance, our study lays the groundwork for further research into combination regimens that prolong drug efficacy without exacerbating toxicity.

With our research as the basis, intricate in-vivo studies can be designed, potentially translating into meaningful clinical benefits. In particular, identifying IL-6 as a key player in sorafenib resistance paves the way for the targeted therapy of resistance mechanisms.

### 5.1. Conclusions

In summary, we demonstrate that low-dose, clinically relevant celastrol reverses acquired sorafenib resistance in HCC cells in vitro, an effect mediated by inhibiting sorafenib-induced IL-6 secretion. Crucially, this resistance reversal is achieved without the hepatotoxicity associated with high celastrol concentrations, highlighting its potential as a safer adjuvant strategy. This work underscores the significance of low-dose celastrol for improving sorafenib efficacy and durability. Key future directions include robust preclinical validation using advanced in-vivo models to confirm efficacy and probe microenvironmental effects, coupled with in-depth mechanistic studies to pinpoint upstream regulators of IL-6 and assess modulation of other resistance pathways. Subsequent pharmacokinetic evaluations and well-designed clinical trials will be essential to ultimately evaluate the therapeutic potential of this combination in overcoming the challenge of sorafenib resistance in HCC patients.

## Data Availability

The dataset presented in the study is available on request from the corresponding author during submission or after publication.
